# Agent Based Model of Anti-Vaccination Movements: Simulations and Comparison with Empirical Data

**DOI:** 10.3390/vaccines9080809

**Published:** 2021-07-21

**Authors:** Pawel Sobkowicz, Antoni Sobkowicz

**Affiliations:** 1 NOMATEN Centre of Excellence, National Centre for Nuclear Resarch, 05-400 Otwock-Świerk, Poland; 2 National Information Processing Institute OPI, 00-608 Warsaw, Poland; a.sobkowicz@outlook.com

**Keywords:** anti-vaccination, vaccine hesitancy, social networks, simulation, agent based model, MMR

## Abstract

**Background**: A realistic description of the social processes leading to the increasing reluctance to various forms of vaccination is a very challenging task. This is due to the complexity of the psychological and social mechanisms determining the positioning of individuals and groups against vaccination and associated activities. Understanding the role played by social media and the Internet in the current spread of the anti-vaccination (AV) movement is of crucial importance. **Methods**: We present novel, long-term Big Data analyses of Internet activity connected with the AV movement for such different societies as the US and Poland. The datasets we analyzed cover multiyear periods preceding the COVID-19 pandemic, documenting the behavior of vaccine related Internet activity with high temporal resolution. To understand the empirical observations, in particular the mechanism driving the peaks of AV activity, we propose an Agent Based Model (ABM) of the AV movement. The model includes the interplay between multiple driving factors: contacts with medical practitioners and public vaccination campaigns, interpersonal communication, and the influence of the infosphere (social networks, WEB pages, user comments, etc.). The model takes into account the difference between the rational approach of the pro-vaccination information providers and the largely emotional appeal of anti-vaccination propaganda. **Results**: The datasets studied show the presence of short-lived, high intensity activity peaks, much higher than the low activity background. The peaks are seemingly random in size and time separation. Such behavior strongly suggests a nonlinear nature for the social interactions driving the AV movement instead of the slow, gradual growth typical of linear processes. The ABM simulations reproduce the observed temporal behavior of the AV interest very closely. For a range of parameters, the simulations result in a relatively small fraction of people refusing vaccination, but a slight change in critical parameters (such as willingness to post anti-vaccination information) may lead to a catastrophic breakdown of vaccination support in the model society, due to nonlinear feedback effects. The model allows the effectiveness of strategies combating the anti-vaccination movement to be studied. An increase in intensity of standard pro-vaccination communications by government agencies and medical personnel is found to have little effect. On the other hand, focused campaigns using the Internet and social media and copying the highly emotional and narrative-focused format used by the anti-vaccination activists can diminish the AV influence. Similar effects result from censoring and taking down anti-vaccination communications by social media platforms. The benefit of such tactics might, however, be offset by their social cost, for example, the increased polarization and potential to exploit it for political goals, or increased ‘persecution’ and ‘martyrdom’ tropes.

## 1. Introduction

The negative societal impact of the anti-vaccination (AV) movement is a very important and urgent subject of research. First, and foremost, this interest is driven by the apparent ineffectiveness of the pro-vaccination efforts of many governments in democratic countries to combat vaccination refusal and reluctance, even during the COVID-19 pandemic. The same problem may be formulated in reciprocal terms: what are the factors driving the persistence of the minority AV opinions and their resilience to rational argument? To preserve the benefits achieved through universal application of mandatory vaccines, such as MMR or polio, we need to understand much better how opposition to vaccination spreads and how can it be countered.

The phenomena related to the AV movement are extremely complex and combine several crucial challenges of modern psychology and sociology: the competition between rational and emotional decision-making, discussion of the role of highly-visible individuals in shaping societal trends, the role of an active versus passive approach to information, the interaction between different factors driving polarization (e.g., between splits in political opinion and a pro-vaccination/anti-vaccination stance), to name but a few. Thus, research on this topic is not only needed, timely and important, it is also challenging scientifically.

At the same time, the AV movement is tightly coupled to general changes observed in social communication and decision-making, namely the steeply increasing reliance on social media and the role of echo chambers, misinformation, fake news, and manipulation (whether by human agents or by AI algorithms). In this sense, the AV movement is a perfect example of the role of the new communication landscape (technological and social) in determining the dynamics of opinion in modern societies, linking the virtual and real worlds.

Even before the COVID-19 pandemic, the AV movement was the subject of numerous studies, spurred by its potentially disastrous consequences and the mystery of resistance to and rejection of rational scientific arguments and the seemingly obvious global benefits of vaccination. There are several general reviews of the literature or comprehensive reports, for example, [1,2,3,4]. The reviews stress the growing importance of the infosphere for the AV movement and the increasing impact and resilience of misinformation spread online on vaccination decisions. It is thus no surprise that a large number of published papers focus on the presence of anti-vaccination arguments on social networks, homophily found in the AV support base, trends in vaccination related Internet searches, selective information processing and other aspects of the online activities of opponents of vaccination [5,6,7,8,9,10,11,12,13,14,15,16]. These infosphere-focused studies are complemented by in-depth research into individual motivation and behavior, for example, [17,18,19,20,21].

Another set of research directions involves the role of media coverage and the question of the spontaneous vs. induced nature of societal vaccination behavior [22,23]; the role of differences in the narratives between pro- and anti-vaccination messaging and associated emotions [6,24,25,26,27,28]; stigmatization of AV activists and its role in entrenchment [23,29]. A separate branch of research is devoted to strategies combating vaccination hesitancy [12,17,21,30,31], the role of communication tools [19], and medical practitioners [2,3].

In this work, we combine large-scale analysis of data covering AV activity on the Internet with an Agent Based Model, describing the behavior of various relevant actors (AV activists, pro-vaccination messaging and health service activities, varied reactions of the general public). By combining Big Data observations with an Agent Based Model, we were able to overcome some of the weaknesses of the data analyses, specifically in the domain of understanding causation, uncovering the hidden dynamics and guiding further data collection. Creation of a successful model of anti-vaccination activism and its influence on societies not only improves understanding of the hidden dynamics of the movement, but could also lead to more effective countermeasures and campaigns, regaining a high level of acceptance of vaccination and, consequently, healthier societies.

## 2. Materials and Methods

As the current work combines two research methods: analysis of large datasets documenting individual online activities related to the AV movement and an Agent Based Model, providing a theoretical framework to explain the observations, the present section is divided into two sub-sections describing these methods.

### 2.1. Dataset Descriptions

To overcome the limitations in temporal resolution in the published literature related to the AV movement, we have undertaken the analysis of big datasets of Internet based communications in which members of the general public play an active role, authoring the texts studied. The data covered the US (Reddit comments) and Poland (a discussion forum related to a Web news site). In both cases, we cover multiyear periods with daily time resolution.

The Polish data are taken from the Interia web site (https://www.interia.pl/, accessed on 1 March 2021). Interia is a news integrator and community portal. Our data are a complete corpus of the user comments posted under news articles. The Reddit data (treated as US representative, although participants may come from any geography) consist of user posts grouped in individual communities called subreddits and discussion threads devoted to specific topics. More than 430 million people are active on Reddit worldwide with over 2 million subreddits. Thus, Reddit can be a significant source of online data, comparable to Twitter and Facebook. The choice of the two sources of empirical data was motivated by a combination of accessibility of complete records and the requirement that the content be (in principle) freely created by the general public (as opposed to traditional media content). It was thus assumed that the comments related to vaccination would serve as a reasonable proxy of the public interest, and, in particular, could be compared with activity modeled in our ABM. In the case of Reddit, the assumption of completeness of the data was only partial. Many comments in the dataset are stored as ‘deleted’, which may reflect active policies of Reddit. Such policies are not visible in the Interia dataset (although some participants complain of deletion or blocking of their posts before they appear).

The whole Interia dataset includes 50,530,059 comments, signed by 3,772,477 unique nicknames. Because the site does not require strict identification of commentators, the actual number of unique users is most likely smaller. The data cover the period from January 2005 to the present time. The Interia web site allows open access in its original form, which has enabled us to download and mine the data via an automated process. Each comment contained the following fields—username (which may be non-unique), timestamp, comment identifier, comment text, and parent comment identifier. This dataset is currently unreleased in its raw form. We have analyzed the dataset searching for all user posts related to vaccination (without distinguishing pro- and anti-vaccination). The search used a set of vaccination related keywords, listed in the Supplementary Material. Comments flagged as related to vaccination were grouped into daily and weekly bins.

The Reddit environment is much more complex than Interia, and poses significant challenges—as documented in [32]. The first challenge is the sheer size of the Reddit community and its activities. The second is the hierarchical organization of Reddit comments, with a broad division into long-term ‘subreddits’ devoted to some general topic, and—within them—user initiated ‘threads’, which have a finite activity span of six months. We focus our long-term analysis at the level of subreddits, where we hope to identify the temporal behavior of vaccination related comments.

The Reddit comments are publicly available as a fh-bigquery dataset, which contains a reddit_comments database (https://bigquery.cloud.google.com/dataset/fh-bigquery:reddit_comments (accessed on 16 July 2021)). This database is divided into tables for a year (for years up to 2010) or year-month (for later years) period and each table contains all comments from Reddit for a given interval of time. Each comment contains at least the following fields—username, subreddit_id, comment id, comment text, and timestamp. Data can be accessed using the Google BigQuery console and queried using standard SQL commands. Access to this dataset is free for the first terabyte each month, with the rest priced according to Google Cloud pricing.

Of course, the two data sources are different in many respects. In addition to the number of posts and users, there are also more subtle differences that impact the data analysis. Because Interia comments are ‘attached’ to specific news items, the comment threads are largely driven by the visibility of the original news story. As the news story drops from the front page, most of the portal users (those who are not specifically interested in the continuation of the discussion) lose the motivation to comment. This leads to a relatively short lifetime of the individual discussion threads which are visible to the general audience. By contrast, the Reddit specialized subreddits, some of which may be much more long-lived, may gather non-overlapping sets of users.

### 2.2. State of the Art in ABMs of the AV Movement

Among the many ways that social phenomena can be investigated, Agent Based Models (ABMs) have been gaining popularity in recent years. While still not fully integrated into mainstream sociology and psychology, they are becoming recognized as useful tools, especially in the context of studying ‘what-if’ scenarios and uncovering causal mechanisms linking various aspects of the studied social system. The ABM framework typically consists of a combination of agents (representing one or more types of social actors, which may be individuals, organizations, or even impersonal entities), the connections between the agents (and between agents and the external world) and agents’ activities and interactions. An ABM model provides a simulation of the studied social system, with qualitative/quantitative accuracy dependent on the model assumptions. The models may significantly vary in terms of the complexity/simplification of the above components, depending on the characteristics of the social system they attempt to describe. The ABM models allow the theoretical hypotheses on which the models are built to be tested. They can also uncover unexpected behavior resulting from interactions. As noted above, the ABM approach allows ‘experimentation’ with various conditions and parameter values, allowing what-if scenario evaluation. Lastly, ABMs can suggest that certain components are missing from theoretical descriptions. Various models have been applied to describe changes in political opinion, diffusion of innovations, economic behavior, and many other social systems. However, despite the importance of the subject and the relative wealth of data, there are only a few examples of models devoted to the study of the AV movement.

The complexities of the mechanisms driving the AV movement described (partially) in the previous section indicate that building a comprehensive agent based model of the AV movement is a challenging task. There are certain classes of general opinion dynamics Agent Based Models which can be tried to explain the persistence of the anti-vaccination movement. Such minority persistence is often explained by inclusion of a very small core of agents with fixed opinions, variously dubbed ‘inflexibles’, ‘fanatics’ or ‘extremists’ [33,34,35,36,37,38,39,40,41,42,43]. The presence of even a small number of such agents often leads to the existence of a much larger minority forming around them. In some cases—especially when the majority is open to discussion, tolerant and lacks its own ‘zealots’—these models show that a tiny ‘contrarian’ part of the population may even ‘convert’ the whole population to their views. Such models reproduce the general observation of the stability of the AV minority observed in democratic societies, but, unfortunately, they lack the depth needed, for example, to model the effects of actions countering the AV propaganda. We shall return to the subject in the next section.

Another class of models of the vaccination debate builds on the variety of the models describing the spread of infectious diseases, for example many variants of the SIR (susceptible-infected-recovered) model, well established in the literature [44,45,46,47]. It is possible to combine the status of an agent with respect to the disease with its status regarding opinions about preventive measures, such as vaccination. This class of models would become very useful in understanding the extreme situation of a massive recurrence of a given disease, but have limited value in the current situation when actual contact with the illness is orders of magnitude less probable than exposure to communications about it.

The difficulty of constructing a comprehensive model of AV behavior is amply documented by the ‘TELL ME’ project, devoted to simulation of decisions taken by individuals in response to an influenza epidemic [48,49]. The model created in the project had considerable complexity, combining numerous processes and parameters. Unfortunately, this very complexity and lack of sufficient understanding of individual contexts and behavior resulted—according to the TELL ME Authors—in an inability to use the model for the planning of strategies in the real world. At the same time, the model had significant development value, allowing a better recognition of the gaps in knowledge and data needs.

Another example of a model dedicated to pro-/anti-vaccination stances was that proposed in [50]. The approach modified Axelrod’s model of cultural dissemination, including dynamical homophily between agents coupling the information flow and epidemic prevalence, via four states (susceptible, exposed, infectious and recovered, SEIR). The model treated public interventions as an external field influencing agents’ behavior.

Another model, combining the epidemiological and information-based factors determining MMR vaccination decisions has been proposed recently in [51]. This model allows the effects of health policies and actual vaccination rates to be studied, since it combines the information flow and epidemiological feedback mechanisms.

The current state of ABM research devoted to the AV movement calls for further effort directed at understanding the nature of the phenomenon, which would preserve the simplicity necessary to understand the model, its assumptions, and starting conditions and yet provide needed insights into ways of effective support for vaccination. In our work, we aim at partially filling the existing model-empirical evidence gap.

### 2.3. Complexity of Applying ABMs to the Anti-Vaccination Movement

The inherently reductionist approach of ABMs, focusing on simplified properties of ‘atomized’ constituents of the studied group and their stylized interactions leading to measurable, large scale phenomena, bears a significant similarity to atomic physics. Unfortunately, most of the current generation of ABMs used to describe the dynamics of opinion changes is too simple to catch the complexity of the opposition to vaccines.

The first source of this complexity is the diversity of the roles and characteristics of ‘actors’ in vaccination treated as a social process. We have already mentioned the ‘practitioners’: doctors and representatives of the authorities responsible for vaccination, including government officials. While we may assume that their task is to promote mass vaccination, there may be situations in which they deviate from this role. A family doctor might not want to enter into discussions or quarrels with his patients, so he/she avoids defending vaccination, or the doctor may have some professionally reasonable doubts resulting from a specific health situation, which are subsequently misunderstood and misused as general doubts about vaccination. There might also be representatives of the medical profession and politicians who *choose* to adopt an anti-vaccination stance as a career move, adding visibility and credibility to the AV movement.

The characteristics of the people taking vaccination decisions (e.g., parents of children who should take the MMR vaccine) are also quite varied and changing in time. The level of education is an example of a non-trivial variable. While one might naively expect that the higher the level of education, the higher the acceptance of vaccination, this is not always the case. As is known from psychology studies, higher knowledge and intelligence may be used to find arguments supporting the current person’s viewpoint, rather than to change it in view of facts that contradict it. This lack of a direct effect of education on the acceptance or rejection of scientific facts has been well documented in the case of climate change [52,53,54]. Kahan [55,56] has shown the motivations behind such an apparent paradox, and suggested that the personal quality necessary to accept views contradicting currently held beliefs is not education or general intelligence but *scientific curiosity* [57]. We note, however, that these findings may be particular to the USA, as a recent study [58] suggests. In the case of the AV movement, higher education and social status may also be connected to a better awareness of the legal ways of challenging the pro-vaccination state system, such as using different forms of legally available exceptions. In addition to education, the population may also be divided into passive recipients of the message permeating the media (official, traditional, or social networks) and seekers of information, often actively filtering and focusing their searches.

On the other hand, a lower educational level may be connected to higher respect for the authority of medical experts, but this is by no means guaranteed. The distrust of government may ‘spill over’ to all activities pushed by it, including vaccination campaigns. Ref. [59] shows that pro-vaccine messages may in some cases actually increase misperceptions or reduce vaccination intentions, depending on preexisting views.

An example of such a situation may be provided by the use of legal coercion and threats of prosecution or loss of privileges (such as exclusion of non-vaccinated children from creche/kindergarten services) for non-compliance with mandatory vaccination. In many cases, this threat (or even the effort needed to get round it) is enough to ensure vaccination. However, in some cases, the legal pressure may result in a rejection, especially if the government regulations are perceived as part of a ‘big pharma’ conspiracy. The same conspiracy accusations weaken or even reverse pro-vaccination communications emanating from medical professionals. As is well known from studies of conspiracy theories and their influence [60,61,62,63], such connections may completely ‘switch off’ any impact of pro-vaccination arguments on certain groups of recipients. This has been analyzed in the context of AV in [25,64,65,66]

Just as positioning of pro-vaccination communicators as ‘big pharma lackeys’ creates distrust, portraying anti-vaccination activists as heroes and martyrs acts to strengthen their message. The ease with which proponents of the two stances can be labeled negatively or positively is especially important for swaying the opinions and decisions of undecided actors. Moreover, this positioning asymmetry is a ‘stable’ one: it is very hard to play the role of a martyr or a hero if you are a government or health official. This difference in emotionally laden labels is an example of a broader trend. In most cases, pro-vaccination communications rely on rational argument, ‘fact-based’, and scientifically supported. On the other hand, the anti-vaccination movement uses emotionally loaded communications, artfully combining various affective components. Starting from parental love and care for children (or natural care for one’s own health), through the stress of the right to take individual decisions, to the creation of fear (made even deeper by the fact that the danger is invisible, and the potential blame—for taking the positive vaccination decision—would lie on the parents’ conscience) and even appeals to religious taboos (such as the vaccine/embryo connections). There is simply no way to compare the emotional ‘arsenal’ of the two sides of the vaccination debate. Okuhara et al. [67] discuss this difference in the context of System 1/System 2 cognitive processing [68] and recommend that pro-vaccine messages should target System 1 in addition to the rational System 2 arguments.

In addition to labeling, we should also take into account the social motivation of the supporters of pro- and anti-vaccination opinion. Most of the people vaccinating their children (or themselves) are simply quiet about the fact. They are a silent majority. There may be many reasons for this silence, but one crucial difference between the vaccinating majority and the anti-vaccination minority may be that the majority treats the debate as unimportant, as part of everyday life, akin to sending children to school or washing one’s hands. The people who vaccinated their children and did not observe any adverse effects are not motivated to rant about it on the Internet, to create grass-roots organizations and to help each other in supporting their views. In many cases, they may return to the issue, when the time comes to vaccinate their grandchildren, if at all.

In contrast, the opponents of vaccination are quite strongly motivated. The reasons for this high involvement may differ and are a powerful combination of positive and negative emotions: love and concern about the well-being of children coupled with fear for their safety, religious views, feelings associated with the beleaguered stronghold mentality. In summary, the range of behavior is by no means a simple pro-/anti-vaccination dichotomy.

Another aspect that must be taken into account in describing communications related to the vaccination debate is how easy it is to convey the pro- and anti-vaccination messages as narratives. Most pro-vaccination content refers to general or statistical arguments (e.g., the ‘need to preserve global immunity’). The AV arguments often use stories about individual tragedies, personal narratives—modes with much greater impact and accessibility, easily transferable between various media: web sites, social networks, face-to-face meetings…

Lastly, to understand and model the details of the evolution of the AV movement, we would have to account for such aspects as the visibility of the actors in the social sphere and the trust in the active supporters of the opposing views. We would also have to take into account effects such as echo chambers, confirmation bias, and modes of communication. The pro-vaccination content originating from medical authorities and governmental agencies is typically impersonal, addressed to populations, not specific individuals. In most cases, it is used in the *push* mode, without active initiation by the recipient. For legal and scientific reasons, the content is complex and contains many caveats (which makes it vulnerable to exploitation by opponents). The trustworthiness of such messaging is highly dependent on the labeling of the sources, which may be discredited quite easily.

The anti-vaccination activists may be divided into two groups, depending on their origin. The first type is a relatively small group of anti-vaccination ‘professionals’. The existence of such a group has recently been confirmed by the studies conducted on Facebook (a Facebook internal study with a report provided in [69]) and Instagram [70]. Among this group, one would certainly count the few members of the medical profession or scientists who oppose vaccination, as well as certain celebrities, movie stars, etc. They see their activities as a global ‘mission’, and if challenged (for example in legal processes, leading to expulsion from the medical profession) adopt a ‘martyrdom’ stance, which actually increases their influence rather than decreases it.

The second AV activist group are people who became afraid of vaccination and not only seek confirmation of their fears but also spread the AV gospel with the zeal of neophytes. They are the majority of active communicators within the AV movement. While they may not be as active as the previous group, their participation in Internet discussion groups or social media, and, especially, their personal contacts create a critical mass. The personal stories serve as proof of the validity of the AV accusations and the universality of their fears. If someone you know expresses doubts and concern, such a stance can be intimately significant for you. The ‘common people’ are motivated and often highly credible—who would doubt the sincerity of a concerned mother? They strengthen the individual appeal of the AV position in ways not accessible to government propaganda.

An important aspect that has been omitted from the discussion so far is the *reality of the illnesses* that the vaccines prevent. The COVID-19 pandemic made this aspect clearly visible. In addition to the AV movement, in most countries, a significant part of society accepted various forms of conspiracy theories doubting the impact (or even existence) of COVID-19, creating memes such as ‘plandemic’. In contrast to many conspiracy theories, the AV movement relates to the potential for actual human tragedy and suffering. A massive scale refusal to vaccinate, breaking the herd immunity could lead not just to localized outbreaks of preventable diseases, but to a return to the tragic state, which most of the western countries have already managed to forget. Some decision makers ‘hope’ that the visibility of a disease would serve as a pro-vaccination communication by itself. However, waiting for such ‘natural’ pro-vaccination arguments is simply immoral, as it is connected with the suffering of many people. Thus, while the ultimate goal of combating the AV movement is medical—building large scale immunity—the ‘battlefield’ for hearts and minds today is the infosphere.

### 2.4. Agent Based Model Description

The model presented in this work includes directly some of the actor and process characteristics described in the previous section, with the goal of addressing three major aspects:

- the increasing, potentially dominant role of social networks and other elements of the infosphere, especially for active information seekers;
- consideration of the emotional asymmetry in information processing;
- the potential for modeling corrective actions (e.g., pro-vaccination campaigns).

The model is written in the NetLogo framework [71,72], which provides a simple, extensible code and integrated graphical interface. This allows other researchers to use and modify the model.

There are four types of agent entities in the model: ‘**doctors**’—representing official communications and ‘real’ doctors, in personal contact with agents. The number of doctors is relatively small (100).

The second category represents the studied population, and we use the name of ‘**patients**’ to denote this category. Note that, in contrast to the growing number of participants in Internet discussions analyzed in our paper, the simulation uses a constant number of patients $N_{P}$—thus we expect the results to correspond to the ‘normalized’ rather than the ‘raw’ data. The presented simulations used $N_{P}=2000$, twenty times larger than the number of doctors.

The third group, very small in number, is called ‘**initiators**’. These are special agents whose opinions are unchanged, and whose ‘task’ is to create the initial stream of persuasive messages. The model assumes that the number of initiators is very small, which is in full agreement with recent studies of AV activity covering Facebook and Instagram [69,70].

The last group of model entities represents the ‘**messages**’ in the infosphere. These messages are created by the initiators and by patients with sufficiently extreme opinions (such patients become ‘activists’). The program uses a fixed number of patients throughout the simulation $N_{P}$ (in the results presented here, this number has been set at 2000).

All four types of agents are characterized by an **opinion** on vaccination, ranging from +2 (strong support for vaccination) to –2 (strong opposition to vaccination). When a new patient is created, its initial opinion is randomly drawn from a truncated normal distribution with the center at $O_{C}$ and standard deviation $O_{SD}$. In the results presented in this work, we have used a symmetrical distribution with $O_{C}=0$ and $O_{SD}=0.5$; these parameters are adjustable via the program interface.

A patient *i*, whose opinion $O_{i}$ is smaller than the **vaccination threshold** $V_{T}$, ‘decides’ not to vaccinate. The results presented here are for $V_{T}=-1$. This choice reflects the assumption that, even for people ‘mildly’ opposing vaccination, the burden of countering legal obstacles necessary to convert their doubts into the actual decision might result in vaccination. The number of agents who do not vaccinate (‘**antivaxxers**’, $O_{i}<V_{T}$) is denoted as $N_{AV}$, and is one of the central measures of the time evolution of the system.

A patient whose opinion is below the **activism threshold** $A_{T}$ becomes an ‘**activist**’ in the AV movement. It joins the initiators group in actively creating anti-vaccination messages. During simulation runs, the number of such activists may become far greater than the number of initiators. The value of the activism threshold $A_{T}$ is one of the crucial parameters determining the behavior of the system. The current number of activists is denoted by $N_{A}$.

The ‘doctors’ opinions are prepared in a special way. The group of doctors is divided into two subgroups: those actively supporting vaccination, and those who—for various reasons—decide to remain uncommitted, or to refrain from communicating their pro-vaccine stance to patients. This reluctance might result from genuine doubts about specific vaccine applicability or from an all too natural desire to avoid heated exchanges or quarrels with a patient. The model assumes that the relative size of the group of uncommitted doctors is proportional to the ratio of antivaxxers among patients: $N_{DU}=kN_{AV}/N_{P}$, where the proportionality factor *k* is assumed to be smaller than one. Thus, for example, k=1/2 means that the ratio of doctors avoiding strong support for vaccination would be half of the current ratio of antivaxxers among patients. The opinions of doctors actively supporting vaccination are drawn randomly from the range [1,2], while the opinions of the uncommitted doctors from the range [−0.25,0].

Messages may be ‘created’ by initiators and activists at each time step of the simulation, via **writing probability** $P_{W}$. Each such message clones the current opinion of its creator. In the case of initiators (whose opinions are fixed at values below the activism threshold), the messages simply repeat their initial opinions. The messages created by activists at different points in time may have different opinion values.

In addition to opinion, patients and messages have a finite lifetime in the system, so each of them is characterized by its age. A note on the meaning of ‘lifetime’ is needed here. This is set not to represent the lifetime of a person in a society, but rather the finite time span of active interest in vaccination. For most people, this is relatively short and limited to the time during which their children are of vaccination age—the time for active vaccination decisions [73]. A nominal lifetime of the patient was chosen as 90 simulation time ticks, without a specific correspondence to the real world time units (at least not before we make comparisons with the observations).

The modal allows for a different ‘aging’ of messages and activists. The rationale behind this flexibility is the relatively short time that messages on the Internet and social networks remain visible or promoted by display and search algorithms. At the same time, with the exception of truly dedicated ‘permanent’ activists (described in our model by the small number of ‘initiators’), the interest of patients turned anti-vaccination activists may be longer or shorter than for typical patients.

### 2.5. Simulation Process

**Figure 1:**
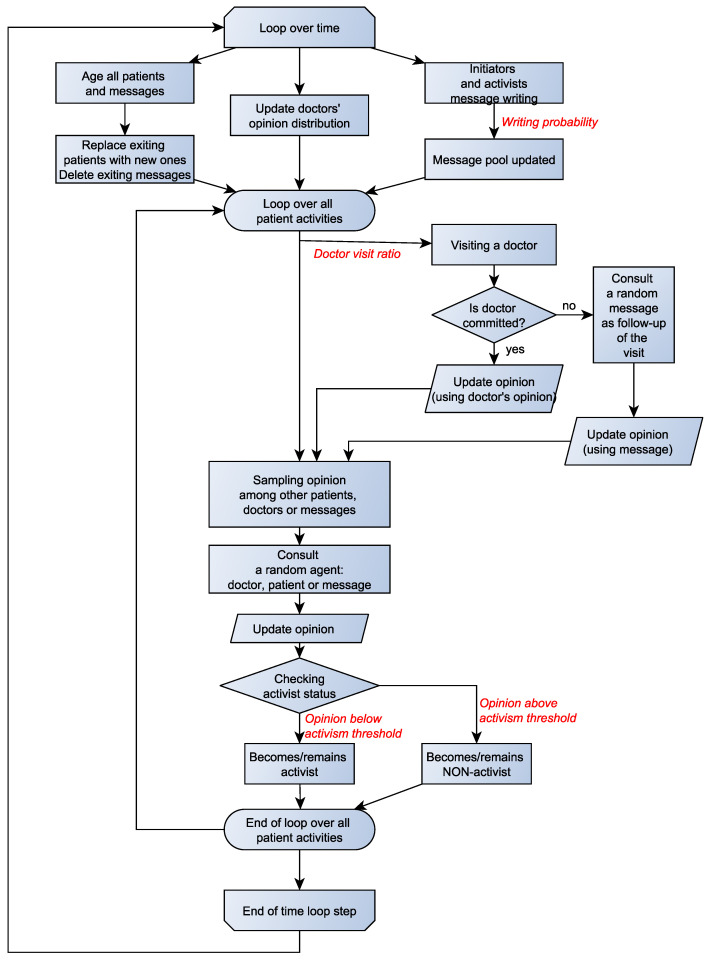
Flowchart of the opinion update process. Red text denotes the key control variables of the simulation.

The flow of the simulation consists of four main processes (the flowchart for the simulations is shown in Figure 1). The first is the life-cycle of patients and messages: their aging and exiting the system when a certain age is reached. In the case of patients, to keep their number constant, each time a patient reaches the maximum age, it is replaced by a new agent, with the initial opinion drawn from the same truncated normal distribution as that used for the original set. When a message reaches the maximum age (set at 90 simulation steps), it is not automatically replaced, which means that the number of ‘active’ messages may vary with time.

The second process is the creation of messages by the initiators and activists. At each time step, each initiator and activist (patient with opinion $O_{i}<A_{T}$) may ‘write up to five messages’—copying its current opinion. Each such writing event happens with a **writing probability** $P_{W}$, which is one of the key parameters determining the results of the simulation. The higher the probability $P_{W}$, the higher the average number of messages written by the initiators and by the current number of activists.

The third element of the simulation flow is a ‘visit to a doctor’. It represents both literal contacts with family doctors, etc. and exposure to official information about vaccination. At each time step, each patient may ‘visit’ a random doctor from the pool (as noted before, divided into committed and uncommitted doctors). The probability of such a visit is the *doctor visit ratio* $P_{VD}$. The outcome of the visit depends on the commitment of the doctor. If the doctor is actively supporting vaccination, the patient modifies its opinion to align with the doctor’s, in a process that shall be described shortly. On the other hand, if the doctor is uncommitted, the patient turns to other sources of information, namely the existing pool of messages. The opinion update algorithm then uses the message opinion as input.

The fourth element represents exposure of the patient to contacts with external sources of information. All active patients participate in the process, in a random order. The source of the information may be a random choice of a doctor, another patient or a message. The message pool includes all active messages in the system, not just those written in the specific simulation turn. Once this source of information (a specific other patient, a message, or a doctor) is chosen, the active patient updates its opinion in accordance with the algorithm described below.

The opinion update algorithm takes into account several peculiarities and biases associated with the emotional asymmetry of the vaccination opinion dynamics. Let us consider patient *i*, with opinion $O_{i}$, encountering information source *S*, characterized by opinion $O_{S}$. The new opinion of patient *i* is given by the transition

(1) $$O_{i}\left(new\right)=O_{i}\left(old\right)\left(1-\alpha f_{iS}\right)+O_{S}\alpha f_{iS}.$$

This form of opinion change is similar to one used in many opinion dynamics models. In plain language, it can be stated as a tendency to adopt a new opinion that is somewhere between the current opinion and the opinion expressed by the source. Here, α is a factor determining the general rate of acceptance—the higher it is, the closer the resulting opinion of patient *i* to the opinion of the source. The value of α is assumed to be the same for all encounters and agents. The value of α=0.66 used in simulation presented in this work supports relatively fast convergence of opinions between the patient and the source.

The current model differs from similar models used previously in opinion dynamics due to the inclusion of bias in information processing. The additional factor $f_{iS}$ describes the filtering of information due to the state of the patient *i* and the difference between its opinion $O_{i}$ and the opinion of the source $O_{S}$. Patients who are relatively undecided (that is those where the absolute value of opinion is lower than the **filtering threshold**$F_{T}$) do not filter information, for them $f_{iS}=1$ regardless of $O_{S}$. On the other hand, when the patient’s opinion is more extreme ($|O_{i}|>F_{T}$), the value of $f_{iS}$ is smaller than 1. For non-activist agents (those with $O_{i}\geq A_{T}$), the values of $f_{iS}$ are given by

(2) $$\begin{array}{ccc} f_{iS} & = & 1\;\mathrm{if}\mathrm{sign}\mathrm{of}O_{i}\mathrm{and}O_{S}\mathrm{is}\mathrm{the}\mathrm{same}, \end{array}$$

(3) $$\begin{array}{ccc} f_{iS} & = & \frac{2-|O_{i}|}{2-F_{T}}\left(<1\right)\;\mathrm{otherwise}. \end{array}$$

In other words, such a patient discounts the opinions of opponents and the more strongly, the more committed it is to the current stance (pro- or anti-vaccination); opinions agreeing with the current one are treated without bias.

For anti-vaccination activists ($O_{i}<A_{T}$), the rejection of less committed opinions is even stronger:

(4) $$\begin{array}{ccc} f_{iS} & = & \frac{2-|O_{i}|}{2-F_{T}}\;\left(<1\right)\mathrm{if}\mathrm{sign}\mathrm{of}O_{i}\mathrm{and}O_{S}\mathrm{is}\mathrm{the}\mathrm{same}, \end{array}$$

(5) $$\begin{array}{ccc} f_{iS} & = & 0\;\mathrm{otherwise}. \end{array}$$

Translating these conditions into plain language, activists discount opinions of sources sharing their stance (indicating their inflexibility); and totally reject sources with opposing opinions. The value of $F_{T}$ determines the effectiveness of the filtering. The results presented in this work assumed $F_{T}=1$ (so that an antivaxxer with opinion $O_{i}<V_{T}=-\left|F_{T}\right|$ would automatically start filtering opposing opinions).

To reflect the special status of anti-vaccination communications (especially in the infosphere), due to their assumed emotional appeal and narrative presentation, we mimic the more universal appeal of **messages** (as the source). This is done by waiving the filtering of their influence, i.e., setting $f_{iS}=1$ for all patients *i* when the source *S* is a message. The only exceptions to this are current activists (who apply the formulae 4 and 5).

During the simulations, non-activist patients and messages age in accordance with the simulation time steps. In contrast, activists are assumed to age at half this pace. This modification of the aging algorithm was chosen to reflect the longer commitment of anti-vaccination activists to the ‘cause’. Instead of losing interest in the issue when their children are past the age of mandatory vaccination, activists may devote their time and effort much longer. This slow-down kicks in when the patient becomes an activist, and returns to the normal rate when it loses this status. Aging of agents is represented by their movement in the program GUI interface.

One of the typical problems encountered in comparing ABMs to real world situations is finding the correspondence between the simulation steps and time scales in empirical observations. This problem is also present here. As shown in Section 3.3, it was possible to calculate the approximate value of the conversion factor between the simulation time and ‘real time’—but the factor turned out to be different between Reddit and Interia data.

## 3. Results

**Figure 2:**
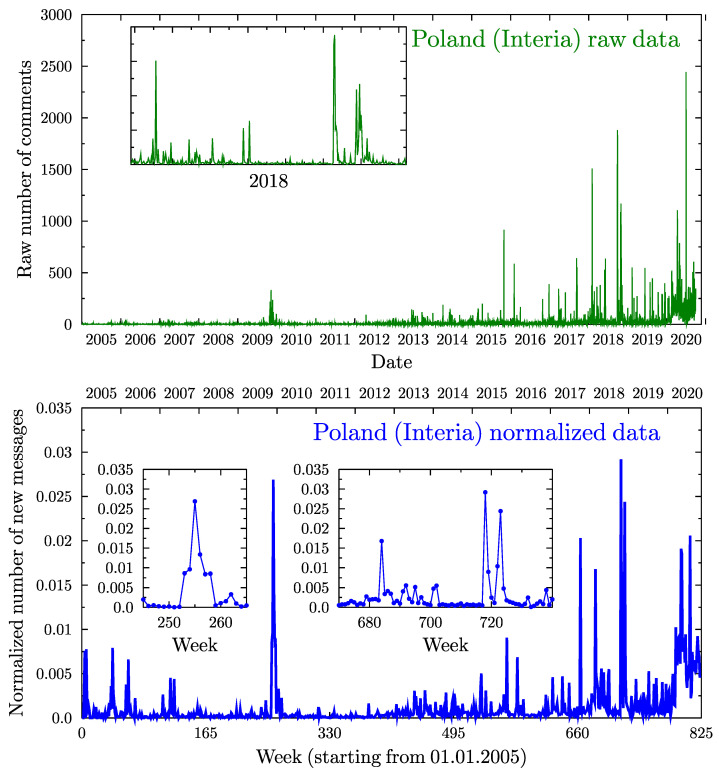
(**Upper panel**): Daily number of new user comments related to vaccination in the Interia dataset, as given by the keyword search algorithms. (**Lower panel**): Normalized number of new user comments appearing on the Interia Web page related to vaccination each week. Because the general traffic at the site has been steadily increasing since 2005, the weekly vaccination related message number is normalized to the smoothed total number of comments at a given time. This allows the number to be compared with the model (which assumes a constant volume of agents and activity). The normalization enhances the role of the observed peaks in activity prior to 2012. One may note the presence of several well defined spikes and a general increase in activity since early 2020, connected with speculations on COVID-19 vaccines.

There are numerous studies of various forms of online activities (user posts, tweets, Google searches…) related to the anti-vaccination movement. Many of these studies detected that these activities appear in well defined bursts. Many of the early studies recording temporal changes of vaccine-related infosphere activity exhibit bursts of rapidly occurring events separated by longer periods of inactivity, noted more than 15 years ago by Barabási [74], and explored in numerous Internet based environments [75,76,77,78,79]. These results were confirmed in more recent works. Mavragani and Ochoa [13] have analyzed the long-term (2004–2017) temporal behavior of Google search intensities related to measles in several European countries (both Western and Eastern Europe). The activity was measured on a monthly basis and for most countries confirmed the spiky nature of the online interest in both English and local language texts. Aquino et al. ([11], Figure 1) presented results for Google searches and Twitter posts, in the context of relevant newspaper coverage for a 6 year period in Italy. Edelstein et al. [80] and Ward et al. [81] provide similar analyses for the UK and France, but without time dependence of the activity. Huang et al. ([9], Figure 2) have compared the number of vaccine related Twitter mentions and CDC vaccination prevalence estimates in the US for a period of three years (2013–2017) with weekly data. Another temporal analysis of Twitter of pro- and anti-vaccination tweets was made by Gunaratne et al. ([14], Figure 2) covering a longer period (2010–2019), but with much more coarse sampling (quarterly). Arendt and Scherr [15] studied the chronology of media attention, public attention, and actual vaccinations during a recent measles outbreak in Austria. The paper focused on a single spike of interest, its origin and effects, especially the decay of media and public attention. Similar spikiness was found by Shin et al. [82] in the case of political misinformation, which shares many psychological mechanisms with the AV movement.

Unfortunately, with the exception of the Huang et al. paper [9], the data are aggregated over relatively long periods (months or quarters), which wash out the fine structure of attention growth and decay (estimated by Arendt and Scherr to be on the scales of single days and weeks, respectively). The smoothing of the data due to their accumulation over longer periods misses the crucial aspect of inherent nonlinearity of the vaccination discussions, due to the presence of numerous feedback mechanisms. For this reason, we focused our analyses on the fine-grained temporal behavior. Our work confirms and extends the accuracy of the papers cited above.

### 3.1. Polish Dataset (Interia Web Site Discussions)

Figure 2 presents Interia comment activity identified as vaccine related (via the keyword search). The upper panel presents the absolute daily number of such comments (‘raw data’). The gradual growth of this number, observed in both the height of peaks of activity and the low activity background, may be traced to the increased popularity of the Web site as a whole. The general traffic at Interia has been steadily increasing since 2005. The average weekly number of *all* comments in the first half of 2005 was approximately 2500, which grew to over 20,000 by 2012 and to over 170,000 by 2020. The traffic increase happened in a relatively smooth manner, which allowed us to normalize the number of vaccination related messages by dividing by the smoothed total number of comments at a given time. Such re-scaling allows us to focus on the relative interest in vaccination related topics. The normalization enhances the role of the observed peaks in activity prior to 2012, and the results are presented in the lower panel of Figure 2 (normalized numbers). The step-like increase since early 2020 (week numbers greater than 800) is related to the COVID-19 pandemic.

**Figure 3:**
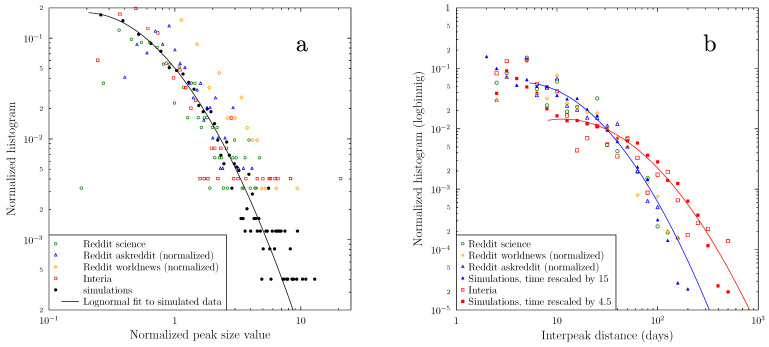
(**a**) histogram distribution of relative new messages peak heights for the empirical datasets and for the simulations (averaged over 20 simulation runs). The line shows a lognormal fit to the simulation data; (**b**) histogram of the distribution of distances between peaks in the same datasets as shown in panel a. Filled symbols show the inter-peak distance distribution in the simulations with simulation time rescaled by 4.5 and by 15 (to fit the data from Interia and Reddit, respectively). The lines show lognormal fits to the simulated data.

There are two crucial characteristics of the observed behavior. Firstly, on the scale of days and weeks, the activity pattern of peak heights and their separation seems random. Secondly, activity waxes and wanes very quickly (on a multiyear scale), but it is impossible to predict the size of a peak from its initial growth rate. Figure 3 summarizes the distribution of the relative peak heights and the time separation between peaks. Both follow roughly log-normal distributions.

A weakness of keyword-based selection of user posts is that it can not reliably recognize automatically whether the post represents a pro- or anti-vaccination stance. To overcome this, we have used human testers to evaluate the sentiment of the posts for several of the largest spikes in interest detected by the automatic search. The results of the evaluation are shown in Table 1. The agreement between the testers was quite high, with a Kappa coefficient (calculated treating the posts which the raters were unable to classify as missing values [83]) equal to 0.77.

In addition to the ratios of pro- and anti-vaccination sentiments, the human-conducted classification of the texts of a part of the Interia corpus has revealed that spikes in activity may be triggered by various causes. Most often, it was a news article appearing on the Interia web site, but sometimes the flurry of increased activity followed a single comment (to an article which had little to do with vaccination). At the same time, many AV comments used either direct references to AV Web sites and information therein or copied such information without specifying the source. Typical AV arguments were often repeated, sometimes verbatim, sometimes in rewritten forms. These forms of self-referencing in AV posts were much stronger than in the pro-vaccination voices, which typically represented isolated calls for reason, pointed out the social benefits and achievements of vaccination, without reference to the sources of the arguments supporting vaccines (we may add here that in quite a few cases, the pro-vaccination champions confused the facts, vaccination history and use, providing ready arguments to their anti-vaccination opponents).

### 3.2. US (Reddit) Data

As in the case of Interia, our approach was based on a simple keyword search of the Reddit database (the list of keywords is given in the Supplementary information). We started by identifying the subreddits which contained the largest numbers of keywords associated with (anti-)vaccination. Such a procedure has to be checked for possible mis-attributions. A perfect example is offered by the use of the keyword ‘MMR’. While in the context of vaccination the acronym is clearly related to the AV movement (through its association with the Wakefield autism scare), the same acronym is used in the gaming communities (where it means Match Making Rating). In the whole Reddit data set, due to the popularity of games related subreddits, the latter use was over four times more prevalent than the medical one. Thus, using a naive keyword search including the ‘MMR’ keyword on the whole Reddit data would yield completely irrelevant results.

**Figure 4:**
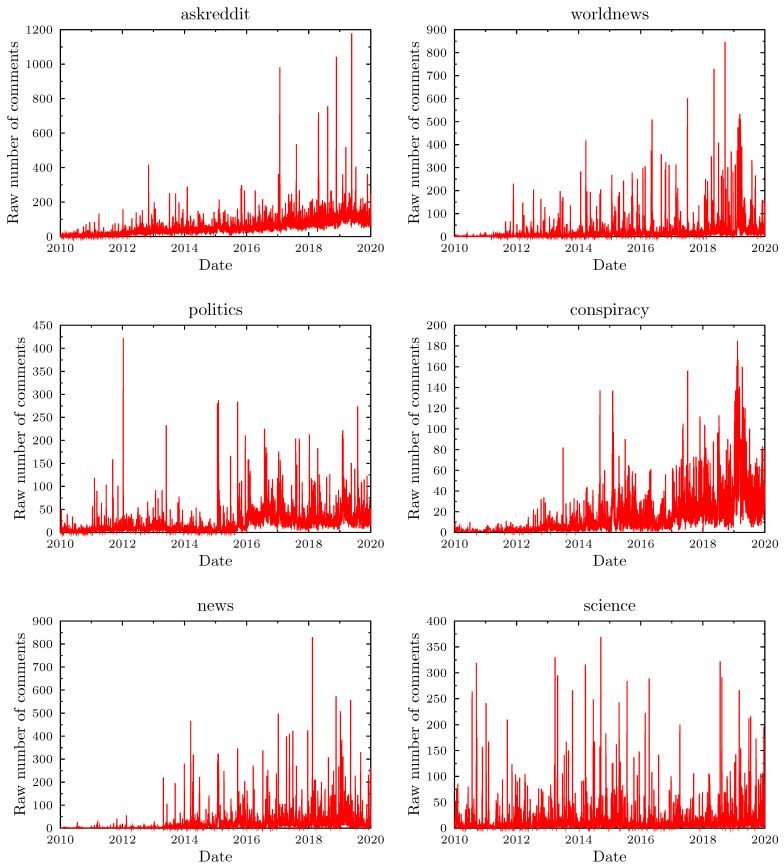
Daily number of new user comments containing vaccination keywords for the six subreddits: *subreddits: askreddit, politics, news, worldnews, consipracy,* and *science*.

The initial analysis, focused on finding the subreddits with the largest number of posts containing vaccine related keywords, resulted in the following selection: *‘AskReddit’, ‘science’, ‘news’, ‘worldnews’, ‘politics’, ‘conspiracy’, ‘todayilearned’, ‘AdviceAnimals’, ‘reddit.com’, ‘aspergers’, ‘funny’, ‘videos’, ‘autism’, ‘explainlikeimfive’, ‘pics’, ‘TumblrInAction’, ‘IAmA’, ‘WTF’, ‘skeptic’, ‘changemyview’, ‘Parenting’, ‘steroids ‘Health’, ‘Drugs*’. Figure 4 shows the time evolution of the number of messages containing vaccine related keywords for the six most relevant subreddits. In all cases, there are sharply defined spikes in the number of posts, but there are significant differences between the subreddits. For example, one observes a steady growth of the ‘background’ level of keyword hits in the *askreddit* case (and, to a smaller extent, in the *news* and *worldnews* cases). The *conspiracy* subreddit also has a relatively higher level of ‘background’ to peak ratio. In contrast, the *science* subreddit shows no significant growth in the ‘background’ and a stable height of the spikes in activity—resembling most closely the normalized Interia dataset.

Five out of the six subreddits shown in Figure 4 (the exception being the *science* one) show a gradual growth in the number of detected posts, both in the peak sizes and in the low activity background. Unfortunately, in contrast to the Interia data, the hierarchical structure and topic-oriented separation of Reddit make the use of a universal normalization impossible. To compare the Reddit results with the simulations (which assume a constant number of patients), we have used ‘normalizations’ limited to specific subreddits.

**Figure 5:**
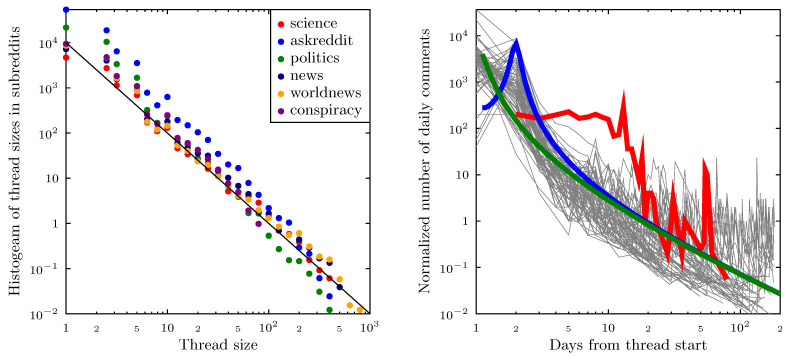
(**Left panel**): Histogram of the overall thread size distribution for threads containing vaccination related keywords within each of the topmost subreddits. The black line shows power–law behavior with an exponent of −2 for comparison. (**Right panel**): Time evolution of the total number of daily comments (not just those containing vaccination related keywords) within the topmost threads containing vaccination related keywords. The time is counted from the day the thread was initiated. For most of the threads, the activity falls very rapidly in the range of 5–10 days, after which typically single comments appear, separated by increasingly long periods of thread inactivity. Green and blue lines show examples of log-Cauchy distributions (green for threads which start ‘immediately’, blue for threads that are ‘noticed’ on the next calendar day.) The red line indicates an example of a thread which is an outlier, showing high and relatively constant activity for over 12 days. The thread, appearing in the ‘*conspiracy*’ subreddit, was titled ‘Vaccines *DO* Cause Autism According to Pro-Vaccine Expert: The sworn affidavit states that he told government officials about the vaccine/autism link long ago, but they kept it secret and promptly fired him’.

The differences between subreddits encouraged us to dig one level deeper, into the threads containing the vaccination related posts. The importance of threads comes from the fact that they are the focus of user involvement, active and passive, at Reddit. To analyze the role of the individual threads identified as containing vaccination keywords, we calculated the histogram of their overall size (all comments within the thread, not just those containing the keywords). The result is shown on a log-log scale in Figure 5 for the topmost six subreddits. Despite significant differences in absolute values, all subreddits show remarkably regular, power-law behavior with and exponent close to −2.

User activity within threads typically decreases rather quickly, falling within 5–10 days to the level of increasingly separated single posts, which may sometimes ‘revive’ the thread long after its origin. The decrease in user activity within threads identified using the keyword search shows weak long-tail behavior and may be approximated by the log-Cauchy distribution. We have identified very few exceptions to this rule (see the discussion in the right-hand panel of Figure 5). Our observations are thus in agreement with [78,84], who showed that the distribution of activity in a subreddit (measured by the time between an original post and the time of the last response) is heavy-tailed, with some posts being active after more than a hundred days. Moreover, Ref. [32] gives examples of subreddits with different authorship characteristics (dominated by original posts, dominated by comments and mixing posts and comments in comparable numbers)—aspects that we also observe.

### 3.3. Simulation Results

This section presents the results of simulations (run in the NetLogo BehaviorSpace framework) conducted in the absence of any pro-vaccination intervention.

While the NetLogo program allows the adjustment of most of the parameters, we decided to focus our attention on a few crucial ones, namely the parameters determining the opinion level at which a patient becomes an activist posting new messages ($A_{T}$), and the probability of writing AV messages ($P_{W}$). The values of the model parameters used in the simulations presented here are listed in Table S1 of the Supplementary Material.

**Figure 6:**
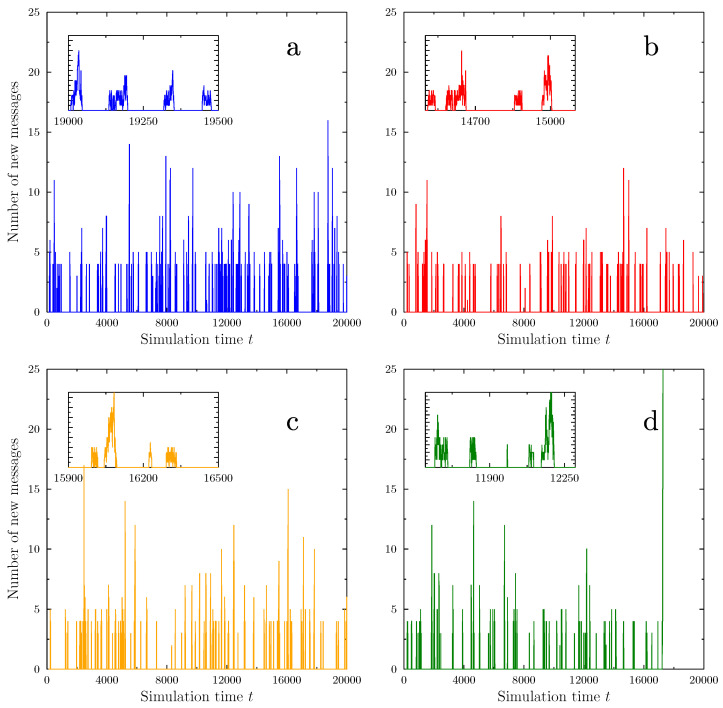
Examples of typical time evolution of the number of new messages written at each time tick of the simulation. The panels correspond to four separate simulation runs, using the same simulation parameters. In all simulations, we observe a series of apparently random spikes in message number. The data exclude messages written by initiators. The insets show close-up views of selected activity spikes. The simulation shown in green (**d**) experiences a transition to a state in which the AV movement triumphs: all agents refrain from vaccination, most of them becoming inflexible activists at transition time t=17,320. The presence of such transitions to a state where the AV movement triumphs depends on the model parameters (such as $A_{T}$ and $P_{W}$, here equal to −1.8 and 0.45, respectively), and the distribution of transition times is close to a power law (Figure 7).

Figure 6 presents examples of the time evolution of the number of new messages appearing in the system during the simulation. The variable parameters used here ($A_{T}=-1.80$; $P_{W}=0.45$) were chosen for the figure because they represent values for which ‘interesting’ phenomena may be observed. Since the activity peaks are very narrow on the total scale of the simulation time (20,000 time steps), the insets show examples of individual bursts of activity. Corresponding figures showing examples of the number of antivaxxers and the number of active messages in the system are presented in the Supplementary Material (Figures S5 and S6 in SM).

Firstly, we observe that the number of new messages is relatively low but exhibits the presence of well defined spikes. During such spikes, a small increase in the number of activists may lead to an increase in the number of AV messages—and consequently an increase in the ratio between the number of instances when a patient ‘consults’ a message versus the number of doctors’ visits. This results in radicalization of AV opinions and may further increase in the numbers of patients refusing vaccination and the number of AV activists. However, as long as the doctors’ influence remains greater than the AV messaging, and the number of activists is small, a random downward fluctuation in the number of activists or the number of AV messages may reverse the trend. As shown in the figure, such situations may repeat many times during a single simulation. This nonlinear mechanism is crucial in creating the spiky nature of AV activity in the model, and provides a suggestion that similar nonlinear feedback mechanisms might be at play in real life.

The distribution of the spikes in the number of new and active messages or the number of antivaxxers, while strongly correlated between themselves for each simulation, seem randomly distributed in size and time, just like the bursts observed in the real-world datasets. Figure 3 compares the distribution of the peak heights for the Interia dataset, three Reddit subreddits (science, askreddit, and worldnews) with the results of simulations. Taking into account that the real-world datasets, despite the effort to use data covering long, continuous times, contain a relatively small number of peaks (which increases the statistical error), the correspondence between the model and observations is rather stunning.

Firstly, the distribution of relative peak sizes is quite similar for the four presented datasets—and almost the same as for the simulation-generated data. All of them follow approximately the same log-normal distribution (panel a in Figure 3).

Secondly, the distributions of separations between activity peaks within subreddits and in the Interia dataset (especially when one considers separations greater than one week, to avoid over-counting) also show statistical regularity. However, the Reddit subreddit data are grouped close to each other, with Interia showing a broader distribution. Remarkably, both datasets can be very accurately approximated by the same simulation results, with the only difference being the scaling between the simulation time step and real world days (Figure 3, panel b). The best fit scaling factors are, respectively, 15 for Reddit and 4.5 for Interia.

The fact that our model was able to reproduce quantitatively the statistical properties of AV activity in two, quite different, infosphere environments serves as significant validation for the model mechanisms and assumptions.

The similarity of the temporal behavior between observational data and the Agent Based Model was quite encouraging. There was, however, a more disquieting observation in the model behavior. For certain ranges of the simulation parameters, a troubling situation occurred. An example is shown in panel d of Figure 6. Instead of shrinking back to the small background level, one of the peaks continued to rise, eventually leading to a global ‘success’ of the anti-vaccination movement: all patients adopting the opinion of the AV activists. Even newcomers, substituting patients exiting the system (assumed to start with neutral opinions), are quickly overwhelmed by their neighbors and by messages. The doctors, faced with such massive opposition, adopt a non-committal state, not wanting to fight the activist majority. The transition to AV stance dominance is extremely rapid on the scale of the simulations (taking as little as 100 time steps—comparable to the ‘lifetime’ of a single patient in the model). Moreover, its initial phase is indistinguishable from the growth of a typical, finite spike. This may cause serious problems from the point of view of containing the AV movement, as the ‘final’, successful push by the antivaxxers may simply be treated as another local activity burst, ‘nothing to worry about’. To understand this dangerous transition, we conducted studies of the conditions under which it may appear.

### 3.4. Modeling Anti-Vaccination Movement Success as a Function of Parameters 

**Figure 7:**
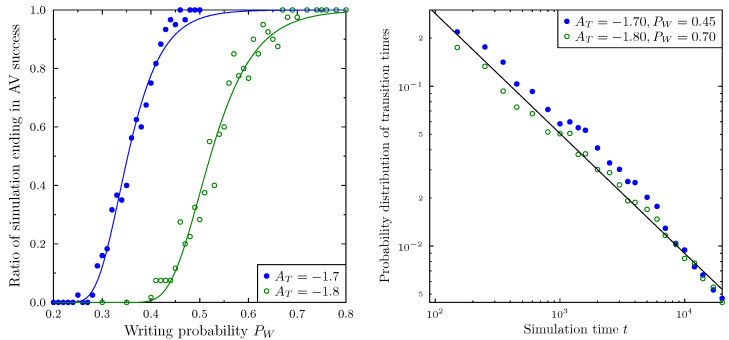
(**Left Panel**): Dependence of the ratio of simulation runs ending in AV campaign success within 20,000 time steps. Point sets correspond to simulations with different activism threshold $A_{T}$. Lines are best fits using a logistic function. (**Right Panel**): Probability distributions for AV dominance transition times for two sets of parameters: $A_{T}=-1.70,P_{W}=0.45$, and $A_{T}=-1.80,P_{W}=0.70$, one thousand simulations for each group of parameter settings. Both cases correspond to situations where most of the simulations result in AV success within the 20,000 time steps limit. Note the log-log scale used. The distribution follows a power law with an exponent close to −0.75 (black line).

The AV success phenomenon present in the ABM displays several interesting statistical properties. It strongly depends on the values of the variables used in this paper: the activism threshold $A_{T}$ and the message writing probability $P_{W}$. Figure 7 presents the ratio of simulations ending in the success of the AV movement. In such a state, most agents become activists and a significant portion of doctors are uncommitted. If we run the simulations for a given value of the $A_{T}$ threshold (below which a patient becomes an activist) and vary the probability of writing, $P_{W}$, the incidence of AV success grows with the increase of $P_{W}$. Above a certain $P_{W}$ value, dependent on $A_{T}$, practically all simulations end with AV success.

The shape of this transition is similar for different values of $A_{T}$, well described by the Gompertz sigmoid function. Decreasing the margin at which patients become activists (e.g., changing $A_{T}$ from −1.7 to −1.8), making it harder to become an activist, shifts the position of the transition to higher writing probabilities.

It is interesting to check the distribution of the transition times in situations when AV success is certain or almost certain. The AV success ‘transition’ does not occur at some pre-determined time; on the contrary, its occurrence seems to be random, with a well defined probability distribution. The right-hand panel in Figure 7 presents this probability distribution for two choices of simulation parameters: $A_{T}=-1.7,P_{W}=045$ and $A_{T}=-1.8,P_{W}=0.7$. Both cases correspond to the range at which AV success is (almost) certain (compare with the left-hand panel of the same figure). In both cases, the probability distribution is well described by a power law with an exponent close to −0.75. It is thus possible that the system remains in a relatively ‘safe’, pro-vaccination state (with only minor peaks of AV activity) for a long time, and then suddenly switches to total vaccination refusal. The randomness of such an event is especially troubling.

## 4. Conclusions

The analysis of Internet based vaccine-related activity presented here—in particular the bursts of active posting and re-posting—draws attention to the nonlinearity in the growth of the AV movement. While our results are limited to just two specific cases (one in the US, one in Poland), they confirm earlier observations of peaks of attention and activity in many more environments. Our study adds fine-grained time resolution, crucial for an understanding of the driving mechanisms. Moreover, the high temporal resolution of our empirical findings shows the true size of the peaks in comparison to the low background in the lull periods, washed out when the data are presented in longer term averages of activity. By combining the detailed observations with an Agent Based Model, we are able to point out the mechanisms behind such burstiness, namely the social mechanisms creating positive feedback loops.

In our opinion, the apparently stochastic, spiky nature of the infosphere based AV communications poses a grave threat to societal health. While at present, in most cases, support for the AV movement is rather low and even at their maxima the spikes do not reach/influence social majorities, the model outcomes show that such situation may be fragile. In contrast to slow changes in opinions, which allow governments and other stakeholders to prepare, apply, and adjust strategic responses, a sharp peak in vaccination reluctance, driven by fear and other strong emotions leave no time for such preparations. The AV activity avalanche predicted by the model might happen too quickly (days, maybe single weeks) to quell anti-vaccination sentiments. The agreement between the observations and the proposed model (despite its obvious omissions and simplifications) suggests that nonlinear mechanisms driven by misinformation, mistrust of authorities, and emotional involvement might push vaccination denial beyond safe limits in a very short time. Moreover, as the observed user activities show, it is hard, if not impossible, to predict which spike in AV interest will grow to what size. This volatility of social reaction to information or disinformation related to vaccines is highly dangerous, as the health authorities are in most cases not prepared to react quickly and adequately.

### 4.1. Modeling Attempts to Counter Anti-Vaccination Movement Propaganda

One of the advantages of ABMs is the ease with which one can modify the simulation code to include, for example, the effects of health authorities’ interventions aimed at stopping the growth of vaccination denial.

These interventions—within our model—can take several forms. One can, for example, increase the ratio of contacts with doctors, $P_{VD}$. We recall here that, in the framework of the model, ‘contacts with doctors’ also includes exposure to all other institutional, pro-vaccination messaging. It is hoped by many authorities that increased contact with pro-vaccination health professionals and official pro-vaccination messages will convince the population and is used in many countries. Unfortunately, such measures disregard the psychological asymmetry of pro- and anti-vaccination communications described in Section 2.3. However, as the spikes in AV activity are driven by nonlinear feedback mechanisms, an increase in pro-vaccination propaganda does slightly decrease the probability of an AV success event, without excluding it. The danger of emotionally-driven massive refusal remains valid. Moreover, an increase in general pro-vaccination propaganda could also have detrimental effects. Requiring more mandatory contacts with health services may be perceived as too burdensome or even as a coercive effort, and could be invalidated via discrediting of the medical services as a ‘pro-vaccination lobby’.

Another method of countering the AV movement is via culling anti-vaccination communications. Such solutions have not only been proposed in academic studies, but also called for by politicians and—in limited scope—implemented by some social network providers [85,86,87]. Our model results suggest that this is the most effective way of avoiding a catastrophic success of the AV movement. Putting a suitable cap on anti-vaccination messages prohibits the explosion of AV sentiment and avalanche-like effects in the model. However, the practice may have unintended negative effects as well. The first problem is the acceptance of censorship as a social engineering tool: once introduced for ‘a noble cause’, who will guarantee the limits of such uses? Second, the AV movement, driven from the ‘mainstream’ social networks and the Internet, would not vanish, but rather assume the mantle of persecuted righteousness, shift to the ‘dark web’, and increase the strength of arguments based on the fight for ‘truth’ against government conspiracies. Modeling the indirect effects of such censorship is beyond the scope of the current model, but should certainly be researched before drastic decisions are taken.

### 4.2. Limitations of the Current Work; Challenges and Directions for Future Studies

The goal of the present work was to create an easily extensible Agent Based Model of vaccination discussions based on an open code framework with embedded visualization and analytical capabilities, and to compare it with empirical findings.

The model presented in this paper contains some, but not all, of the key components of the social system defining the AV movement—for example, stressing the role of the infosphere and its characteristics in AV propaganda, the potential impact of the positions of health workers and medical professionals, as well as the effects of biased assimilation/rejection of arguments and the influence of emotionally loaded narratives. There are several important characteristics that are missing from the current version of the model. Among these model weaknesses, one should mention the absence of a social network structure (present in the AV movement and in most forms of social communication); lack of strong homophily in interpersonal contacts (only partially modeled through rejection processes present in the model); and the use of the same fixed lifetimes for all agents and messages (instead of more realistic distributions). To implement these improvements, it is necessary to gather more detailed empirical information regarding the dynamics of AV communications. Such work is currently in progress.

Another direction of model development is related to modeling the situation created by the COVID-19 pandemic. We stress here that the model described in our paper was formulated with the goal of reproducing the growth of the AV movement and its temporal dynamics in pre-COVID times. In our opinion, the pandemic has significantly changed the conditions driving the activities of the anti-vaccination movement and the popular reaction to these activities.

The impact of the pandemic on everyday life and the economy is significant on a global scale. The combination of fears related to health with the severe effects of social and economic lock-downs creates an emotional landscape in which distrust of the medical industry in general and anti-vaccination arguments in particular can easily take hold. Moreover, the rush with which the COVID-19 vaccines were developed, the demanding conditions for their transportation and storage, and problems with their accessibility and distribution open up a way for AV campaigns using rationally sounding arguments and appeals to safety and good practice. The situation is worsened by the fact that, in most countries, the reactions of governments and health authorities to the pandemic were, to say the least, less than optimal. In many countries, there were successive government blunders, as the authorities tried to balance health concerns with economic analyses. Wild swings between periods of lock-down and loosening of restrictions signaled, to many of us, a lack of coherent strategies. This, in turn, created easy targets for anti-establishment propaganda, facilitating attacks on the health authorities (including vaccine preparation and distribution processes). These attacks—in contrast to the cases of vaccines developed for well known diseases—often focus on actual issues and problems, not just ‘pure’ misinformation. Of course, misinformation is still present in the current AV campaigns, even in its extreme forms (referring to COVID-19 as a ‘plandemic’ or suggesting that the goal is global depopulation or domination via mysterious ‘chips’ embedded in the vaccines).

For these reasons, to construct an adequate model of the AV movement related to the pandemic, we would need to wait for the relevant data to accumulate. This work is in progress. It is our hope to develop an extended model taking into account the lessons learned (so painfully) during the COVID-19 pandemic.

## Figures and Tables

**Table 1:** Ratios of anti- and pro-vaccination posts analyzed by human raters for some of the prominent peaks of activity detected by the keyword search algorithm. The table also contains the ratio of posts for which assignment was impossible.

Spike Date	ANTI	PRO	UNKNOWN	Number of Posts
November 2009	43%	26%	31%	919
October 2015	64%	21%	15%	788
February 2018	73%	17%	10%	1848
October 2018	63%	24%	13%	1058
November 2018	64%	23%	14%	2900

## Data Availability

The source code of the NetLogo ABM used in this work is available at github https://github.com/pawelsobko/AntiVaxxNetLogo (accessed on 16 July 2021) repository.

## Supplementary Materials

The following are available online at https://www.mdpi.com/article/10.3390/vaccines9080809/s1, Figure S1: Normalized number of ‘active’ comments available on the Interia Web page related to vaccination, assuming the lifetime of the comment to be 4, 8 or 16 weeks, Figure S2: Periodogram of the Interia data, using frequencies derived from weekly time stamps, Figure S3: Top panel: distribution of the ratio of posts identified by the keyword search to the overall thread size as function of the thread size for the topmost threads within the six main subreddits, Figure S4: Graphical interface of the NetLogo simulation program, with main components marked, Figure S5: Examples of typical time evolution of the number of patients with opinion below the vaccination threshold (antivaxxers), Figure S6: Examples of typical time evolution of the number of active messages in the system, Figure S7: Periodograms of the simulated number of active messages, using the abstract simulation fre-quencies scaled by a factor of 4.5, Table S1: Parameters used in simulations. References [88,89,90,91,92,93,94] are cited to in Supplementary Materials.

## Abbreviations

The following abbreviations are used in this manuscript:

ABM	Agent Based Model(s)
AV	Anti-Vaccination
