# Supplementary material for: Agent Based Model of Anti-Vaccination Movements: Simulations and Comparison with Empirical Data
Source: Vaccines (Basel). 2021 Jul 21;9(8):809. doi: 10.3390/vaccines9080809 (PMC8402338; doi:10.3390/vaccines9080809)
Supplement: Supplementary file 1 [file vaccines-09-00809-s001.zip › Antixaxx-mdpi-v3-SupplMat-1.pdf]

Supplementary Materials: Agent based model of anti-vaccination movements: simulations and comparison with empirical data

Pawel Sobkowicz and Antoni Sobkowicz

July 20, 2021

1 Supplementary information: dataset analysis

1.1 Vaccination related post search

The vaccination-related comments were selected from the complete datasets using simple keyword searches. The selection of the keywords was based on an initial human-based reading of partial datasets, which allowed identification of terms related to vaccine hesitancy. Al already noted, in the case of the Reddit dataset, the 'MMR' keyword had to be excluded from the search list, due to the popularity of its games-related meaning.

1.1.1 Interia dataset search keywords

Words and word stems used in the Interia keyword search: *'kosikowski','nop', 'zięba', 'czajkowska-majewska','rtęć','szczepionk', 'autyzm','mengele','zaczip','justna soch', 'autyst*', 'MMR', 'Wakefield', 'szczepi*'*

1.1.2 Reddit dataset search keywords

Words used in the Reddit keyword search: *'wakefield', 'vaccine side effects', 'vaccine adverse effects', 'vaers', 'depopulation', 'immunization', 'vaccine', 'mealses', 'pharma', 'pharmaceutical', 'mmr', 'mumps', 'autism'.*

1.2 Additional data analysis results

Within the Agent Based Model, the patients have access to the whole set of active messages, not just to the ones created in the current time tick. This condition was implemented to reflect the real life conditions: people searching for vaccine related topics or browsing past discussions have access to historical posts, not just the current ones. To estimate whether such 'history' based visibility also exhibits spiky nature, we have estimated, for the Interia data, the number of 'visible' comments, assuming that the posts have limited 'screen

lifetime'. Figure S1 presents the time evolution of the number of 'live' messages for lifetime assumed to be 4, 8 and 16 weeks. While such integration process broadens the peaks of interest and washes out small fluctuations, the general nature of randomly distributed peaks of activity remains clearly visible.

The newly posted comments may be transformed to estimate the number of 'active' messages, readily accessible/visible to the Interia users. This is done by assuming that messages have a finite visibility lifetime. In the analysis, we set the lifetime at 4, 8 and 16 weeks, to observe the effects of the comment long-term visibility. The results are presented in the Supplementary Materials.

The randomness of spike sizes and times, while visually appealing, is difficult to assess quantitatively. To provide somewhat better measure of the randomness we have used periodogram analysis. Periodograms capture key properties of the random spikes in activity (peak heights and separation between them) and can provide means to compare the empirical data with simulations. Figure S2 presents the normalized spectral density for the newly posted comments, as well as for the three accumulated, 'active' comment counts spanning periods from 4 to 16 weeks.

An interesting observation related to Reddit individual subreddits and threads containg vaccine-related keywords is the ratio of the posts identified by the search algorithm to the total size of these threads. This ratio varies significantly between threads. Figure S3 presents this ratio from two points of view. It decreases as function of the overall thread size, with roughly power-law decrease with an exponent of -0.67. The longer the thread, the smaller, on average is the proportion of posts identified by keywords. On the other hand, when we focus on the number of keyword containing posts the best fit line is a constant value. For threads with the number of keyword posts between 200 and 600 there is a large variation of the calculated ratio, but above for threads with the number of keyword containing posts greater than 600 the ratio remains close to the 10% for all such threads. At present we have not identified possible mechanisms for these observations.

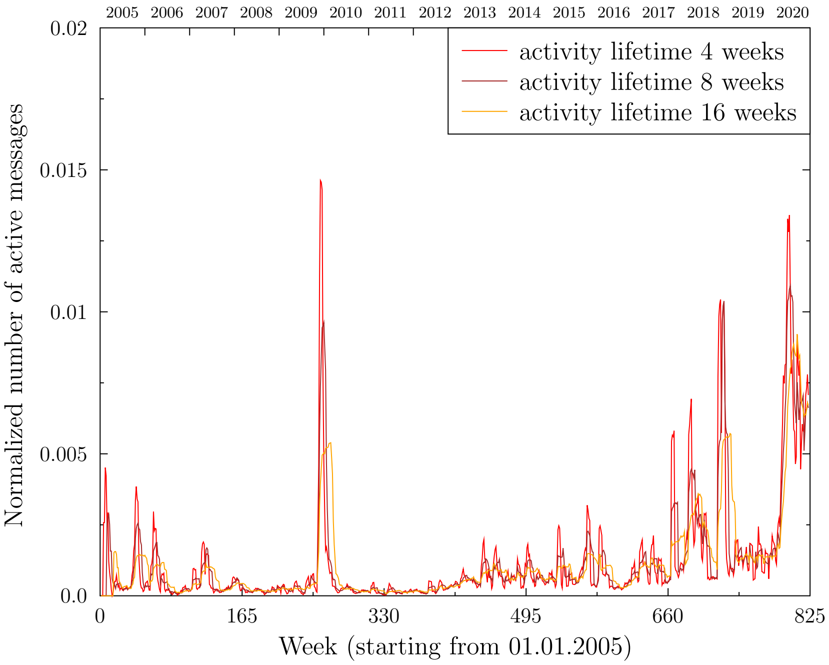

Figure S1: Normalized number of 'active' comments available on the Interia Web page related to vaccination, assuming the lifetime of the comment to be 4, 8 or 16 weeks. Normalization similar to the one used in the bottom panel of Fig. 2 in the main text has been applied. Short retention times (4 and 8 weeks) preserve the peak structure, while the 16 week span washes the details out. With increased lifetime the peaks become broadened, decreasing short-term fluctuations, which impacts the periodogram calculations (shown in Figure S2).

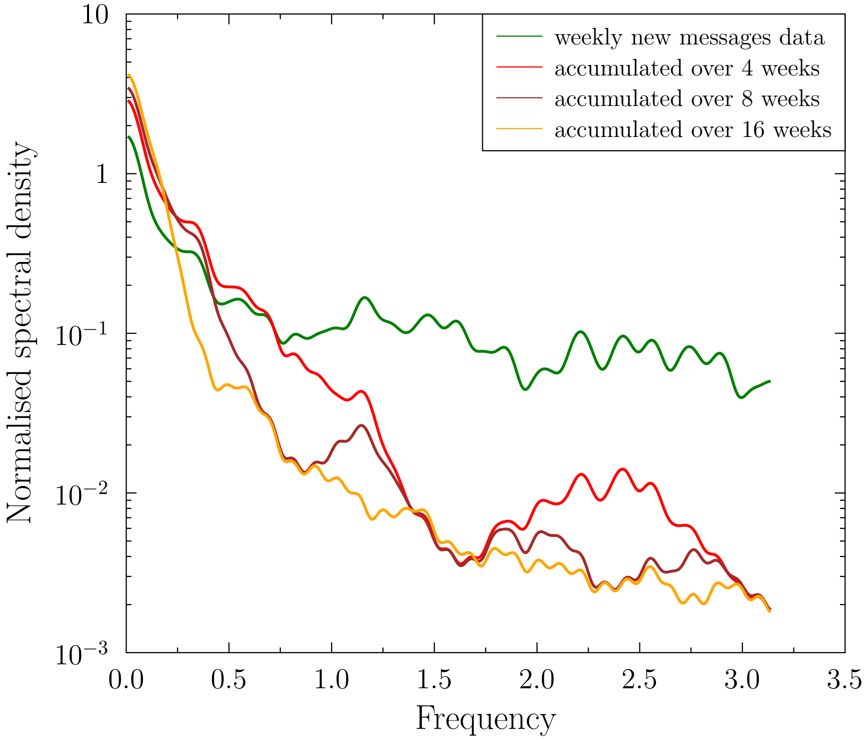

Figure S2: Periodogram of the Interia data, using frequencies derived from weekly time stamps. Green line corresponds to the new messages, red, brown and orange ones to accumulated messages over 4, 8 and 16 weeks.

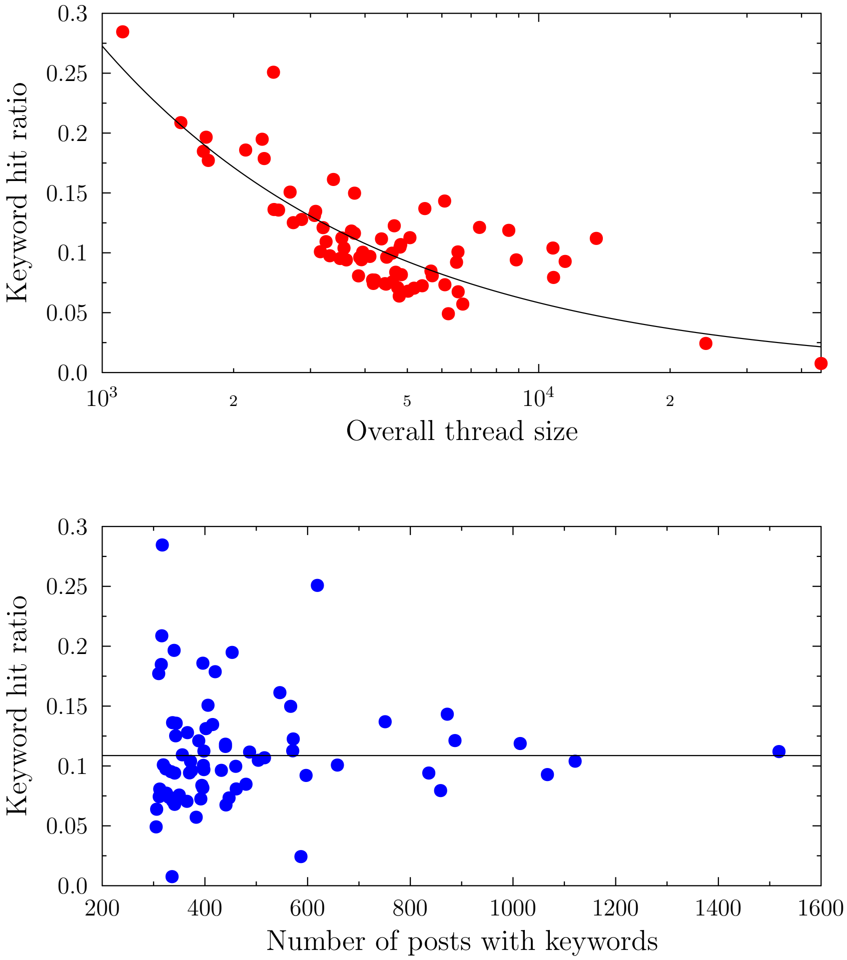

Figure S3: Top panel: distribution of the ratio of posts identified by the keyword search to the overall thread size as function of the thread size for the topmost threads within the six main subreddits. Black line is a fit with power-law distribution with exponent of -0.67. Bottom panel: the same ratio as function of the number of posts identified by the search algorithm. Black line is the best linear fit, which turns out to be constant.

2 Supplementary information: Agent Based Model

2.1 Model NetLogo interface and code

The model code is available at the github repository `https://github.com/pawelsobko/AntiVaxxNetLogo` The program user interface is depicted in Figure S4, while the parameters used in the simulations presented in this work are listed in Table S1.

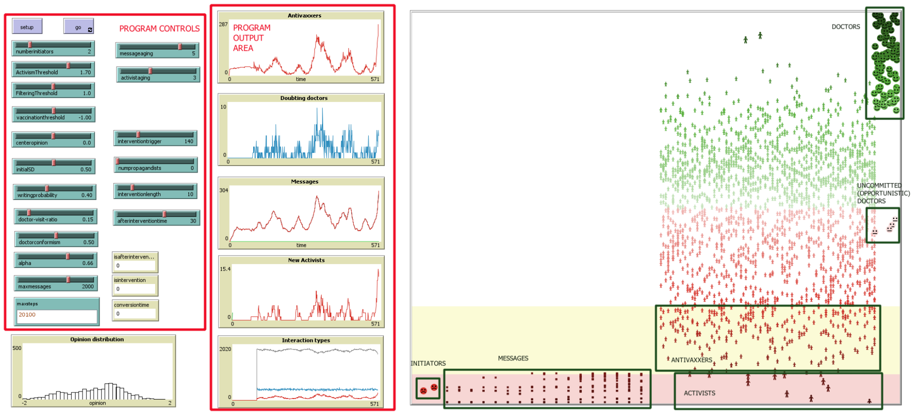

Figure S4: Graphical interface of the NetLogo simulation program, with main components marked. Left side – program control and output area, right side –graphical view of agents: patients, messages and doctors.

2.2 Model results

Figure S5 presents examples of the time evolution of the number of antivaxxers corresponding to the Figure 6 in the main text. Figure fig:exampleMcum shows the results for the number of active messages in the system for the same simu-lations.

Both the observed real-world data and the model show apparently random, spiky temporal dependence of the vaccine related activity. To compare the model and observational periodograms we have to take into account the lack of direct connection between time flow in the model and in reality (in other words, the unknown mapping of the timescale or frequency scale between the two periodograms). The normalized spectral density of the Interia 'active' messages with the model results shows very high resemblance if the time rescaling factor is set at 4.5 (Figure S7).

With just one fitting parameter (the timescale/frequency modification) the statistical properties of the random process described in the model and the observed data in the Interia dataset are remarkably close – especially if one

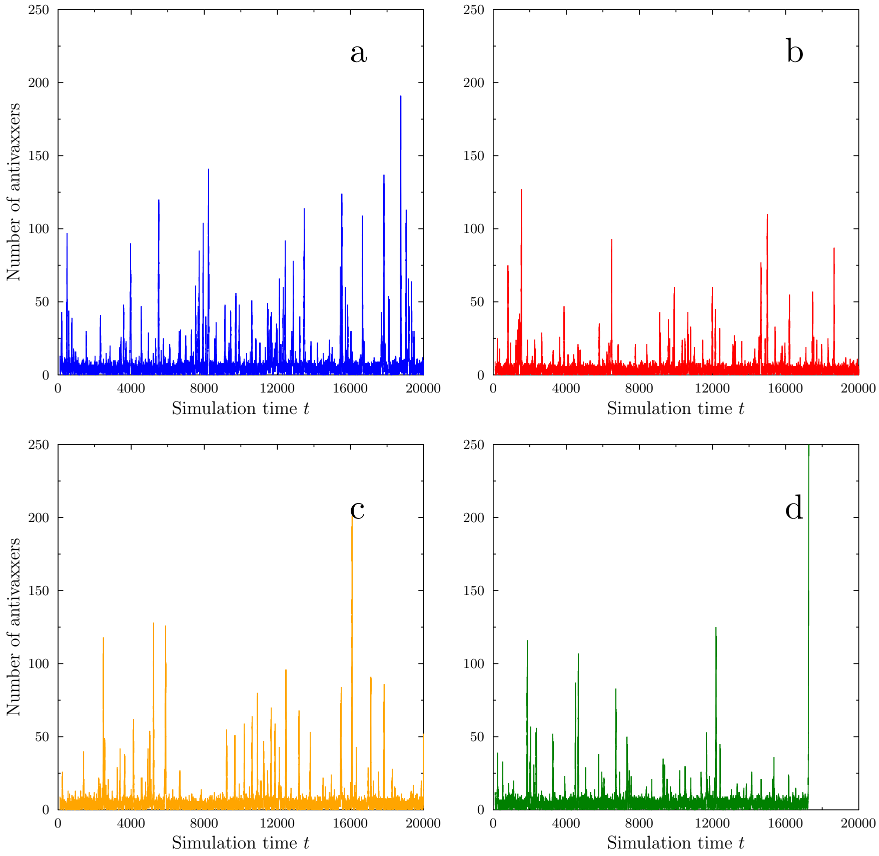

Figure S5: Examples of typical time evolution of the number of patients with opinion below the vaccination threshold (**antivaxxers**). The panels correspond to four separate simulation runs, using the same simulation parameters. In all simulations we observe a series of apparently random spikes in AV activism. The simulation parameters are the same as for Figure 6 in the main text.

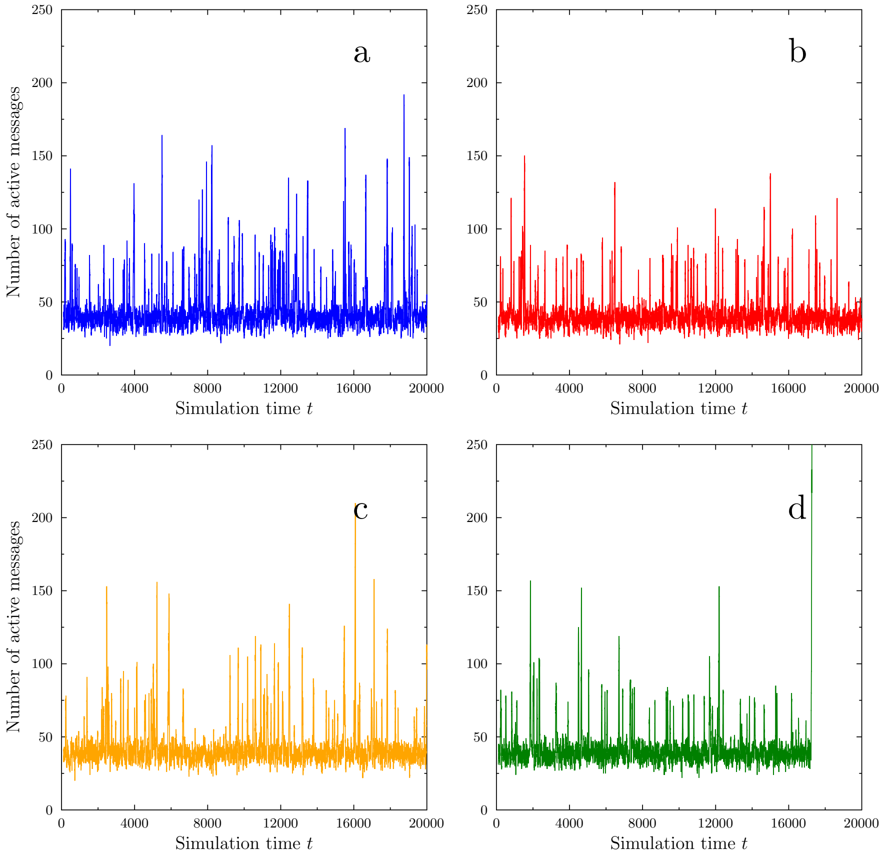

Figure S6: Examples of typical time evolution of the number of active messages in the system. With the aging speedup of 5 used in the simulations, each message remains active in the system for $18 = 90/5$ time ticks. The panels correspond to the same simulation runs as figures 6 in the main text and S5. In all simulations the spikes in active message number shown are broadened but retain the random structure of occurrence times and sizes. The constant background are the messages created by the initiators.

Table S1: Parameters used in simulations. The results presented in this work are controlled by two main variables: A_T and P_W.

Parameter	Type	Value
Vaccination threshold V_T	fixed	-1
Activism threshold A_T	variable	-1.7 to -1.80
Writing probability P_W	variable	0.20 to 0.80
Number of initiators	fixed	1 or 2
Maximum number of messages	fixed	2000
Convergence factor α	fixed	0.66
Conformism of doctors	fixed	0.5
Doctor visit ratio	fixed	0.15
Filtering threshold F_T	fixed	1
Number of patients N_P	fixed	2000
Aging speedup factor for messages	fixed	5
Aging speedup for activists	fixed	3
Maximum age (time ticks)	fixed	90
Centre of initial and new patient opinion distribution	fixed	0
SD of initial and new patient opinion distribution	fixed	0.5

recalls the limitations of the model. Moreover, we can attempt to use the frequency rescaling to try to map the timescales of the data and the model. The patient 'lifetime' assumed in the model (90 time steps) would correspond to $90/4.5 = 20$ weeks time. At the same time the message 'lifetime' (for which we have used a 5 times speed-up factor) would correspond to 4 weeks – exhibiting surprising consistency with the 'best fit' accumulation timescale for the comment lifetime.

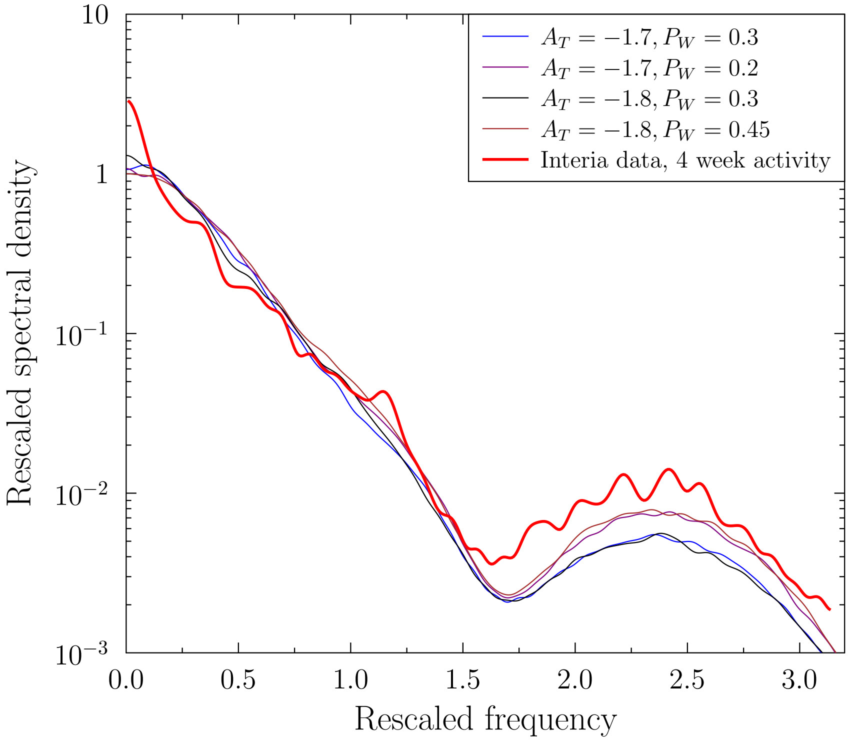

Figure S7: Periodograms of the simulated number of active messages, using the abstract simulation frequencies scaled by a factor of 4.5. The scaling was used to maximize the similarity between the simulated and observed periodogram of the Interia data for the 'comment lifetime' of 4 weeks data (compare Figure S2).

3 Supplementary information: COVID-19 related research

3.1 COVID-19 pandemic

The COVID-19 pandemic has provided a fertile ground for the AV movement. This prompted several studies devoted to various aspects of the 'infodemic' – as the flurry of both accurate and false information related to the pandemic has been called by WHO. Due to the review and publication cycles most of the works cited below describe 'snapshots' of still developing situation, and most were prepared before the actual vaccines were approved, and long before the vaccination campaigns begun. In this sense, many of their conclusions were wither proposals for guidelines for ensuring vaccination support or attempts to predict potential risks ([1, 2, 3]).

Jamison et al. [4] analysed the acceptance of reliable/unreliable information sources in COVID-19 discussions. Interestingly, they have found that while the AV activists shared the greatest proportion of unreliable sources (35.4%), the same proportion for pro-vaccination supporters was a non-negligible 11.3%.

The political connections of the pro- and anti-vaccination voices in the context of the US politics were studied in [5]. Both papers noted an apparent AV correlation with the Republican and Trump support. It is worthwhile to note that [5] observed spiky behavior of online activity, similar to the one reported here (on a daily scale, but for short-term data).

Sturm and Albrecht [6] studied the COVID-19 conspiracy theories and their role in creation of emotional and irrational atmosphere preparing the grounds for vaccination resistance. Similar conclusions were reached by Newhagen and Bucy [7]. To address such potential problems, Young et al. [8] proposed five classes of social network interventions, intended to disrupt the misinformation dynamics. Some of these interventions may be possible to be integrated in an extended version of the current model, offering an ABM framework to test their effectiveness. The rejection of specific COVID-19 vaccines, based on reports of their side effects, blown-out far beyond the actual danger, is a case in point. In many cases (e.g. blood clots for the Astra-Zeneca and Johnson & Johnson vaccines) the health authorities reacted in a way that was confusing to the general public and paved the ground for the AV propaganda. The danger posed by rapid, massive reactions to anti-vaccination stimuli is made even greater in the light of recent reports of disinformation campaigns conducted not only organically by anti-vaxxer activists, but also systematically by professional sources ([9]).

4 Additional References

References

[1] Brunson, E. K., Schoch-Spana, M., A social and behavioral research agenda to facilitate Covid-19 vaccine uptake in the United States. *Health security*, 18(4):338–344, **2020**. DOI:https://doi.org/10.1089/hs.2020.0106

[2] Gayle, H., Foege, W., Brown, L., and Kahn, B., editors. *Framework for equitable allocation of COVID-19 vaccine*, volume 10. The National Academy Press, Washington, DC, **2020**. (https://www.ncbi.nlm.nih.gov/books/NBK564091/, accessed July 15, 2021)

[3] Stolle, L. B., Nalamasu, R., Pergolizzi, J. V., Varrassi, G., Magnusson, P., LeQuang, J., and Breve, F. Fact vs fallacy: the anti-vaccine discussion reloaded. *Advances in therapy*, 37(11):4481–4490, **2020**. DOI:https://doi.org/10.1007/s12325-020-01502-y

[4] Jamison, A. M., Broniatowski, D. A., Dredze, M., Sangraula, A., Smith, M. C., and Quinn, S. C. Not just conspiracy theories: Vaccine opponents and pro-ponents add to the Covid-19 'infodemic' on Twitter. *Harvard Kennedy School Misinformation Review*, 1(3), **2020.** (https://misinforeview.hks.harvard.edu/article/not-just-conspiracy-theories-vaccine-opponents-and-pro-ponents-add-to-the-covid-19-infodemic-on-twitter/, accessed July 15, 2021)

[5] Evanega, S., Lynas, M., Adams, J., Smolenyak, K., and Insights, C. G. Coronavirus misinformation: quantifying sources and themes in the covid-19 'infodemic'. *JMIR Preprints*, **2020**. (https://allianceforscience.cornell.edu/wp-content/uploads/2020/10/Evanega-et-al-Coronavirus-misinformation-submitted_07_23_20-1.pdf, accessed July 15, 2021)

[6] Sturm, T., and Albrecht, T. Constituent Covid-19 apocalypses: contagious conspiracism, 5g, and viral vaccinations. *Anthropology and Medicine*, 28: 122–139, **2021**. DOI:https://doi.org/10.1080/13648470.2020.1833684

[7] Newhagen, J. E., and Bucy, E. P. Overcoming resistance to Covid-19 vaccine adoption: How affective dispositions shape views of science and medicine. *Harvard Kennedy School Misinformation Review*, 1(6), **2020**. (https://misinforeview.hks.harvard.edu/article/overcoming-resistance-to-covid-19-vaccine-adoption-how-affective-dispositions-shape-views-of-science-and-medicine/, accessed July 15, 2021)

[8] Young, L. E., Sidnam-Mauch, E., Twyman, M., Wang, L., Xu, J. J., Sargent, M., Valente, T. W., Ferrara, E., Fulk, J., and Monge, P. Disrupting the Covid-19 misinfodemic with network interventions: Network solutions for

network problems. *American Journal of Public Health*, 111: 514–519, **2021**. DOI:https://doi.org/10.2105/AJPH.2020.306063

[9] Jemielniak, D., and Krempovych, Y. AstraZeneca vaccine disinformation on Twitter. *medRxiv*, **2021**. DOI:https://doi.org/10.1101/2021.04.08.21255107
